# Sexual epigenetics: gender-specific methylation of a gene in the sex determining region of *Populus balsamifera*

**DOI:** 10.1038/srep45388

**Published:** 2017-03-27

**Authors:** Katharina Bräutigam, Raju Soolanayakanahally, Marc Champigny, Shawn Mansfield, Carl Douglas, Malcolm M. Campbell, Quentin Cronk

**Affiliations:** 1 Department of Biology, University of Toronto Missisauga, Mississauga ON, L5L 1C6, Canada; 2 Saskatoon Research and Development Centre, Agriculture and Agri-Food Canada, 107 Science Place, Saskatoon SK, S7N OX2, Canada; 3 Department of Biological Sciences, University of Toronto Scarborough, Toronto, ON M1C 1A4, Canada; 4 Department of Wood Science, University of British Columbia, 4030-2424 Main Mall, Vancouver BC, V6T 1Z4, Canada; 5 Department of Botany, University of British Columbia, Vancouver BC, V6T 1Z4, Canada; 6 Department of Molecular and Cellular Biology, University of Guelph, Guelph ON N1G 2W1, Canada

## Abstract

Methylation has frequently been implicated in gender determination in plants. The recent discovery of the sex determining region (SDR) of balsam poplar, *Populus balsamifera*, pinpointed 13 genes with differentiated X and Y copies. We tested these genes for differential methylation using whole methylome sequencing of xylem tissue of multiple individuals grown under field conditions in two common gardens. The only SDR gene to show a marked pattern of gender-specific methylation is PbRR9, a member of the two component response regulator (type-A) gene family, involved in cytokinin signalling. It is an ortholog of *Arabidopsis* genes ARR16 and ARR17. The strongest patterns of differential methylation (mostly male-biased) are found in the putative promoter and the first intron. The 4th intron is strongly methylated in both sexes and the 5th intron is unmethylated in both sexes. Using a statistical learning algorithm we find that it is possible accurately to assign trees to gender using genome-wide methylation patterns alone. The strongest predictor is the region coincident with PbRR9, showing that this gene stands out against all genes in the genome in having the strongest sex-specific methylation pattern. We propose the hypothesis that PbRR9 has a direct, epigenetically mediated, role in poplar sex determination.

The majority of flowering plants are cosexual (hermaphrodite or monoecious), in marked contrast to animals, which tend to have unisexual individuals. However some 6–7% of flowering plants do have separate sexes (i.e. are dioecious)^1^. These instances of dioecy are scattered across flowering plant clades, and dioecy in plants has many separate origins from an ancestral cosexual condition. Flowering plants are therefore a promising group in which to study the origin and mechanisms of sex determination and the development of sex chromosomes^2^,^3^,^4^,^5^. Some recent progress has been made recently in understanding the diverse molecular mechanisms of sex determination in flowering plants. In monoecious plants a single factor can be key in the development of unisexual flowers, as in melon (*Cucumis melo*), where an ethylene biosynthesis gene is the key determinant^6^. In monoecy, flowers of different sexes are present on the same individual. When dioecy evolves from monoecy, as in *Diospyros* and *Populus*, the determinant of floral sex must become associated with a segregating genomic region, thus becoming a sex locus. In *Diospyros* a small RNA is a likely candidate for the key determinant^7^. In *Populus* the functional molecular basis has yet to be elucidated.

Sexually specific patterns of methylation have frequently been implicated in plant sex determination^8^,^9^,^10^,^11^. In the genus *Populus* (poplars and aspens) methylation has also been implicated in work using the Chinese white poplar (an aspen relative), *Populus tomentosa* Carrière^12^.

The first detailed characterization of the sex-determining region (SDR) in *Populus*^13^ pinpointed 13 genes in a compact non-recombining region with a XX/XY architecture. This SDR has the same genomic architecture in *P. trichocarpa* Torr. & Gray, *P. balsamifera* L. and *P. deltoides* W. Bartram ex Marshall, but is different in aspen (*P. tremuloides* Michx)^13^,^14^.

To test the hypothesis that sex-specific methylation patterns may be involved in the vegetative-phase establishment of sexual differentiation in *Populus*, we wished to examine whole genome methylation patterns of vegetative tissue (xylem) in relation to sex and in particular the methylation status of all the genes identified at the *Populus* sex-locus by Geraldes *et al*.^13^. Xylem was chosen as it is a consistent tissue type with very little phenotypic plasticity (unlike leaves).

## Results

### PbRR9 is the only gene in the *P. balsamifera* genome to show strong sex-specific methylation patterns

Overall we detected expected amounts of DNA methylation across the genome. Taking all the samples (Table 1, S1) together we studied over 49 trillion potential cytosine methylation contexts (CG,CHG, CHH) of which 16.7% were methylated (Table S2). Of the methylated positions genome-wide, roughly equal numbers were in CG, CHG and CHH contexts (30.5, 32.3% and 36.7% respectively; Table S3).

**Figure 1:**
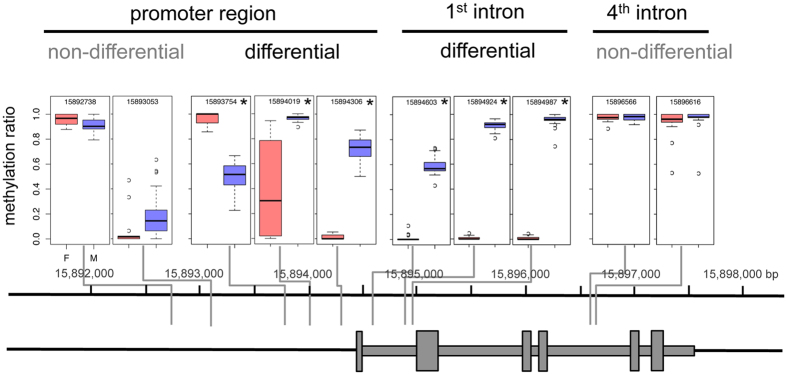
Map of CG methylation across the PbRR9 gene (Potri.019G133600). Pink = female; blue = male. There is strong sex-specific methylation in the putative promoter region and in intron 1. In addition there is strong non-sex-specific methylation in intron 4. Asterisks indicate a significant difference between male and female samples (Q < 0.001, logistic regression test (Akalin *et al*.^42^), n = 18–24, methylation difference >20%).

**Figure 2:**
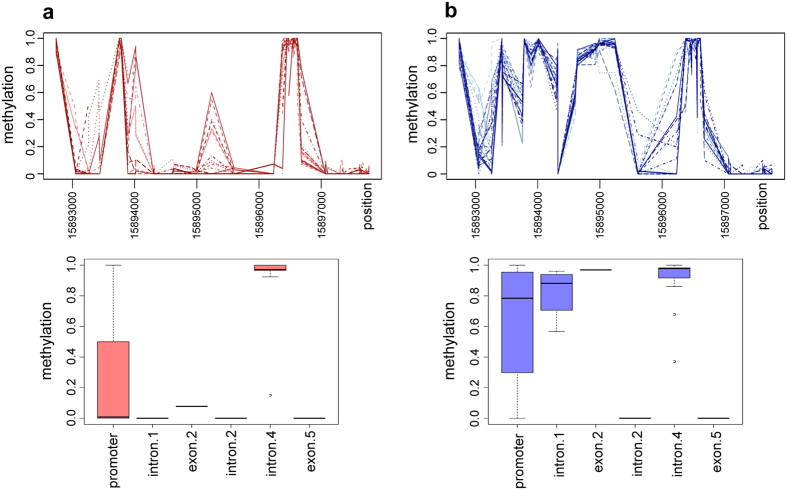
Methylation trends (CG context) in males and females: (**a**) female, (**b**) male. Top: multiple line graphs for males and females revealing a sex-specific “fingerprint”. Bottom: methylation characteristics of various gene features.

**Figure 3:**
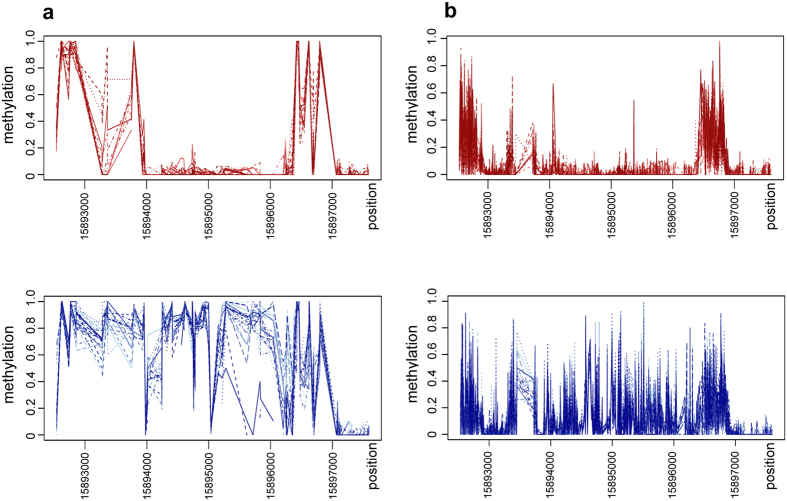
Methylation trends (CHG and CHH contexts). Multiple line graphs for males and females, revealing a sex-specific “fingerprint” in other DNA methylation contexts: (a) CHG and (c) CHH.

We next examined all the genes identified at the sex locus by Geraldes *et al*.^13^ for patterns of sex-specific methylation (Figs S1–3). Only one of these genes showed a distinct pattern of sex-specific DNA methylation: the two component response regulator 9 (PbRR9, Potri.019G133600). PbRR9 is the apparent ortholog of the two *Arabidopsis* genes ARR16 (AT2G40670) and ARR17 (AT3G56380). All these genes are members of the type-A subfamily, which is generally considered to form part of the network transducing cytokinin signals^15^,^16^. We determined that PbRR9 has a marked and significant pattern of differential DNA methylation between the sexes for all methylation contexts (Figs 1, 2 and 3). Figure 2 gives a more detailed breakdown specifically for the example of the CG context. The sex-specific methylation patterns were consistent across the two different environments (northern and southern common gardens) and thus appears to be constitutive.

**Figure 4:**
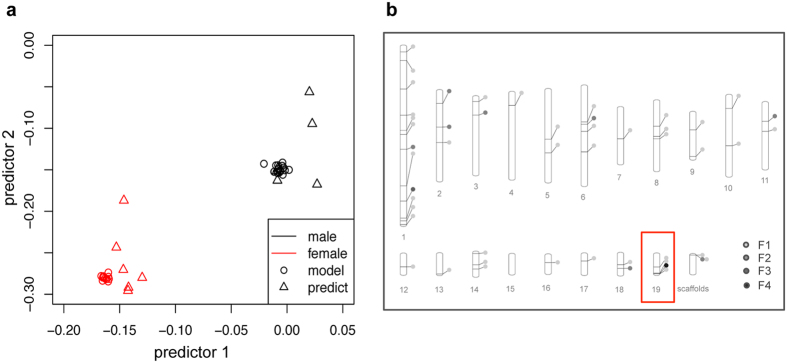
Prediction of gender from genome-wide DNA methylation patterns. Left: Using statistical learning, gender (red: female, black: male) of test individuals (triangles) was assigned accurately on the basis of a training set (circles). The two-dimensional projection of the DNA methylome data shows group centroids of the predictors 1 and 2 for the discrimination of male and female samples on the x- and y axes. Right: To assess robustness and stability of the gender classification, the statistical learning algorithm (PLR) was run multiple times (5 fold cross validation, 50 iterations). Genomic regions whose methylation signatures were selected for modelling are visualized in the graphical representation of the genome (19 chromosomes of the poplar genome, all scaffolds were concatenated into a single unit). The shade of the color indicates the frequency with which features were repeatedly included in the PLR model for gender prediction (F4 > 80%: F3: ≤80%, F2: ≤20%, F1: ≤10% of all meta-features). The darker the marker, the greater the utility in prediction. The only marker in black is the one marking the region of the genome coincident with the PbRR9 gene. This plot shows the results for CG methylation: CHG and CHH contexts show nearly identical results.

We also surveyed sex-specific DNA methylation genome-wide both directly (data not shown) and by using machine learning (see below) to predict gender from genome-wide methylation patterns. We performed this analysis separately for all DNA methylation contexts. Although relatively weak signals of sex-specific methylation are detectable from across the genome (1376 features) the region of the genome coincident with PbRR9 gives by far the most prevalent contribution to the predictive signal (Fig. 4, right). This clearly indicates that PbRR9 is the gene that shows the strongest signal of sex-specific methylation pattern under our experimental conditions.

### Strong sex-biased DNA methylation is characteristic of the promoter and intron 1 regions

Looking at DNA methylation across the PbRR9 gene, some striking patterns are immediately evident (Figs 1, 2 and 3). First, this gene is apparently both “promoter methylated” and “gene body methylated” (i.e. the methylation is in sites of the potential promoter region and in transcribed parts of the gene, mostly the introns), Methylation can be strong in the proximal region close to the transcription start site showing up to 90% methylation (Figs 1, 2 and 3). In addition, methylation occurs in the first and the fourth introns to an extent that is higher than the genome average for intron regions^17^,^18^. Secondly, in general the males are more heavily methylated than the females (Figs 1, 2 and 3), although there is one cytosine of the putative promoter region where this pattern is reversed. The regions of greatest sex-specific methylation bias are the putative promoter region and intron 1 (the latter has strong male-specific methylation). The concentration of methylation at the 5-prime end of the gene is consistent with control regions generally being found in the first intron^19^ and 5-prime to the gene (promoter region). In *Arabidopsis* only about 5% of expressed genes are methylated at the proximal promoter^20^,^21^, but promoter methylation is well known to influence transcription^22^.

### DNA Methylation patterns can be used to assign genders by machine learning algorithms

Knowing the sex of individual poplar trees has allowed us to determine a strong sex bias in methylation at a single locus. Now we ask the question, can knowing the genome-wide pattern of methylation allow us to determine tree gender? Using 70% of the samples as a training set (Tables S1 and S4), the methylation patterns of pseudo-unknown trees were then used to predict gender. With over 200,000 input features (see Methods), a set of meta-features, i.e. feature groups whose collective methylation patterns are highly informative, were useful as predictors and comprised ca. 60 individual features (Table 2). Figure 4 (left) shows that accurate assignment to the correct gender cluster was easily achieved from genome wide methylation pattern. A large number of genome regions were weakly predictive of gender from their methylation, although some of these may represent random effects or small partial contributions due to the large number of genome regions tested. However, one region was very strongly predictive of gender: the sex-determining region on chromosome 19. On this chromosome the number of recurrent predictive features, is considerably higher than on other chromosomes, especially when taking chromosome length and cluster prevalence (37 vs. 3–9, Table 3) into account. This is where gene PbRR9 is located (Fig. 4, right), and PbRR9 has the strongest differential methylation signal of all genes in this region. This confirms PtRR9 as a standout gene, among all the c. 45,000 poplar genes, whose methylation is strongly predictive of gender.

## Discussion

### Is sex in poplar controlled epigenetically?

As the SDR on chromosome 19 is the only part of the genome that segregates with gender, a gene or genes in this region must ultimately control gender. Geraldes *et al*.^13^ first characterized this region, and on the basis of a genome-wide association study (GWAS) using version 2.2 of the genome (the version of the genome that assembles the sex locus least poorly) determined 13 genes at the SDR. One of these genes was methyltransferase 1 (MET1), a cytosine methyltransferase. The presence of this gene at the SDR raises the possibility that sex-specific DNA methylation mediated, in some unknown way, by the male (Y) allele of met1 might be responsible for sex-specific methylation of genes in any part of the genome. However now that sex-specific methylation has been examined we know that the strongest signal of such methylation is at the SDR itself, PbRR9. We therefore have an SDR with a sexually differentiated methyltransferase and a sexually differentiated methylation target, raising the possibility that sex-biased methylation of PbRR9 might be controlled in *cis* or *trans* or both.

Our study was highly targeted in that it looked for sex-specific DNA methylation in a vegetative tissue (xylem) with no sexual phenotype. This is to test the hypothesis that sex-specific methylation patterns may be involved in the pre-reproductive establishment of sexual development. It would be of interest to examine sex-specific methylation in other tissues including reproductive ones. However, the problem of using reproductive tissue is that they differ in physiology, biochemistry and morphology between sexes, so differences in methylation may be due to the sex of the individual, or merely to tissue differences between the tested organs. Testing a non-sexually differentiated tissue allows determination of constitutive sex differences and establishes that DNA methylation is a marker of sex in tissues that have no phenotypic indications of sex, i.e. the methylome precedes the phenotype as a marker of sex.

### PbRR9 is potentially a master regulator of poplar gender

The sex-specific methylation patterns of PbRR9 could be coincidental or responsible merely for secondary sexual characteristics of little relevance to sex determination. However, this gene has a number of features that make a direct role in sex-determination plausible. Type-A two component response regulators are a moderately-sized family of transcriptional activators with a variety of developmental effects^23^,^24^,^25^,^26^,^27^ and so could plausibly trigger a developmental cascade leading to alterations in inflorescences. A previous study in poplar have shown that PbRR9 is only weakly expressed in vegetative tissue but is strongly expressed in catkins^25^ but only female catkins were tested in that study. Many RR genes are known to be part of cytokinin signal transduction. Cytokinins are involved in early inflorescence development^28^ and may thus be part of the inflorescence-specific activation of PbRR9.

The hypothesis that PbRR9 is directly involved in poplar gender determination clearly deserves to be tested further, especially by detailed expression studies of this gene against the background of reproductive development. A fine-scale study of differential expression against the trajectory of inflorescence development, involving microdissection of inflorescence primordia, would be ideal. It is however, beyond the scope of the present paper. Identifying downstream targets of this gene and assessing the phenotype of plants in which this gene has been misregulated, for instance by VIGS or CRISPR/Cas9 genome editing, would also be obvious possibilities.

### Could tree sex be reversed by demethylation?

The finding of sex-specific patterns of DNA methylation raises the possibility that inflorescence sex could be altered by hypomethylating chemical treatment, such as by 5-azacytidine^29^ or zebularine^30^. Such an approach has been applied in *Silene*^9^ in which application of 5-azacytidine (5-azaC) induced a sex change in a number of male plants, while having no effect in females. Such induced sex changes, even if limited to single inflorescences, have some significance for plant genetics and breeding as they potentially allow selfing, which is not normally possible in dioecious trees. It has long been known that sex is labile in poplars: a number of cases of naturally occurring intersexes have been observed^31^,^32^,^33^. Given the results reported here, the possibility arises that some of these intersexes may result from hypomethylation mutations. As far as we are aware, as yet no attempts have been made to induce sexual abnormalities in poplar with chemical hypomethylation, but the idea is open to test.

## Methods

### Source of *P. balsamifera* tissue samples

DNA methylation patterns can vary according to genotype and environmental conditions and so careful replication was used in the experiemntal design, with multiple genetic individuals and multiple sites. The material sequenced consisted of genomic DNA from xylem tissue collected from multiple genotypes in replicate from two environmentally divergent common gardens^34^. The common gardens are located at Prince Albert (PA - 53.62°N 106.43°W; elevation 461 m) and Indian Head (IH - 50.52°N 103.68°W; elevation 605 m), Saskatchewan, Canada and comprise provenances of *P. balsamifera* that were collected throughout the natural range of this species. Two common gardens were used to ascertain the effect of between-site differences in environment on the methylation patterns observed. A fully randomised design was used for the common gardens to eliminate any within-site systematic environmental effects on phenotype or epigenome. The collection of *P. balsamifera* genotypes used here, the AgCanBaP collection of the Agriculture and Agri-Food Canada (AAFC), has been previously described^35^. Genotypes were sexed phenotypically at flowering, or by using genetic sex-tests as described in Geraldes *et al*.^13^. The list of genotypes (and environment of origin) used is given in Table 1 (and further details in Table S1).

Tissue was harvested in the field from small branches of *P. balsamifera* (c. 1–2 cm diam.) from the north-exposed side of the tree. The bark was peeled, and the soft tissue inside (the external layer of the wood cylinder, developing xylem) was scraped off with a razor blade and immediately stored on dry ice. Tissue was harvested on five consecutive days in the field at the end of July in 2013 (PA: July 25^th^ and 26^th^; IH: July 27–29). For consistency, tissue was harvested only during the first half of the day (10 am–2 pm). Xylem was chosen as it is a consistent and homogeneous tissue that has little phenotypic plasticity with environment (unlike leaves).

*P. balsamifera* is the sister species of the fully sequenced *P. trichocarpa* which has been the subject of extensive variation studies^36^,^37^. There is very little difference between the species at the molecular level and there is no difference in the architecture of the sex determining region^13^. Therefore *P. trichocarpa* gene annotations are used throughout.

### Bisulphite sequencing

Genomic DNA was extracted according to Doyle and Doyle^38^ and subjected to whole genome bisulfite sequencing (WGBS). WGBS was performed essentially as described in Lister *et al*.^39^. Libraries were generated using a target insert size of 300 bp and original Illumina adapters. Paired-end sequencing was performed on a HiSeq2500 (Illumina) at 125 cycles per end and by multiplexing 2 samples per lane at the Genome Science Centre, Vancouver British Columbia, Canada.

### Analysis of DNA methylation patterns

Quality and adapter trimmed reads^40^ were mapped to the *P. trichocarpa* reference genome (v3.0, http://phytozome.net) using the three-letter aligner Bismark v0.12.5^41^. Reads were mapped against *in silico* bisulfite converted and non-converted genome sequences, and methylation ratios for each cytosine position were determined as #Cs/(#Cs + #Ts). Downstream processing and calculation of differential methylation was done in R for CG, CHG, and CHH contexts separately^42^,^43^. Pre-filtering included coverage-based selection for bases with coverage >10×. Differential methylation between male and female samples was calculated, and in CG context features with differential methylation of at least 25% and q < 0.001 were selected^42^.

### Sex prediction from DNA methylation by machine-learning

For statistical modeling of methylation patterns on gender, methylation information was summarized across the genome for tiles (features) of 500 bp length. Filtering for statistical learning included selection of variable features with higher than median inter-quartile range that were detected in 80% of the samples, i.e. features with little variation across samples were excluded. This retained a total of 243197 features for use in supervised classification. Penalized logistic regression (PLR) implemented in program pelora^44^ has been developed as an effective means of classification in microarray analyses and was shown to demonstrate excellent predictive potential^44^,^45^,^46^. The pelora classifier integrates feature selection, supervision and sample classification using a forward selection approach with recurrent pruning steps and a l_2_-penalized negative log-likelihood function^44^. Importantly, it identifies groups of features, i.e. clusters or meta-features that collectively contribute to robust outcome variable prediction. Here, PLR was employed to model gender on DNA methylation data. The predictive potential was evaluated using 5 fold cross validation with 50 iterations across 30 randomly selected xylem samples that comprised the training set (random selection by genotype, split1, Table S1). An alternative training set selection (completely random, split2) yielded comparable results. Suitable parameters were selected to identify the most parsimonious model with accurate prediction potential (Table S4). Independent model evaluation was done using separate sets of test samples (Table S1). Leading meta-features were then merged for detailed downstream analyses.

## Additional Information

**How to cite this article:** Bräutigam, K. *et al*. Sexual epigenetics: gender-specific methylation of a gene in the sex determining region of *Populus balsamifera. Sci. Rep.* **7**, 45388; doi: 10.1038/srep45388 (2017).

**Publisher's note:** Springer Nature remains neutral with regard to jurisdictional claims in published maps and institutional affiliations.

## Supplementary Material

Supplementary Information

## Figures and Tables

**Table 1:** List of poplar (*P. balsamifera*) genotypes from the AgCanBaP collection used in this study, with sex indicated.

Sex	Genotypes
Female	BOY12, POR04, POR14, ROS15*, SOU01, SOU03, SOU09, WHR11, WOL08
Male	BOY08, FRE05, FRE12, LAR05, LOV01, LOV4, LOV7, POR05, POR06, POR12, ROS01*, WHR15

Genotypes marked * are *P. balsamifera x P. deltoides* hybrids. All genotypes were replicated by sampling from clones planted in two contrasting environments (Prince Albert and Indian Head, Saskatchewan) except for POR05 and POR12 (Indian Head only). A detailed sample description is given in Table S1.

**Table 2:** Statistical Modelling of gender using CG methylation ([1] = mean ± SD, K = 5, R = 50 iterations).

# Total input features CG context	# Features in predictors[1]	# Average features per cluster[1]	Misclassification rate[1]
243,197	60 ± 11.3	6.0 ± 1.9	0.016

The average number of features that collectively comprise the predictors in PLG to model and predict gender is given along with the number of features per predictor and the misclassification rate in the training set (5 fold cross validation, R = 50. K = 5, λ = 1/32, q = 10).

**Table 3:** Predictor composition in PLR, based on chromosomal location.

Chromosome	# Features[1]	# Unique features[1]	Rel. cluster prevalence[2]
Chr1	14.1 (1.85)	5.8 (0.87)	9.1
Chr2	3.3 (1.40)	1.9 (0.51)	6.0
Chr3	2.5 (0.85)	1.8 (0.59)	4.5
Chr4	2.4 (0.83)	1.9 (0.58)	3.7
Chr5	2.1 (0.66)	1.6 (0.55)	3.5
Chr6	2.8 (0.90)	2.2 (0.54)	4.6
Chr7	1.2 (0.68)	1.0 (0.44)	3.5
Chr8	1.6 (0.84)	1.0 (0.45)	5.1
Chr9	0.7 (0.37)	0.6 (0.29)	4.3
Chr10	1.5 (0.68)	1.3 (0.49)	3.1
Chr11	2.3 (0.78)	1.8 (0.52)	4.8
Chr12	1.1 (0.55)	0.9 (0.41)	3.7
Chr13	1.7 (0.66)	1.4 (0.48)	4.2
Chr14	1.9 (0.81)	1.3 (0.52)	5.0
Chr15	1.0 (0.63)	0.8 (0.36)	3.4
Chr16	1.1 (0.50)	0.9 (0.40)	3.8
Chr17	1.2 (0.64)	1.0 (0.45)	3.3
Chr18	1.7 (0.61)	1.2 (0.43)	4.8
Chr19	11.1 (0.83)	2.0 (0.43)	37.1
scaffolds	4.2 (1.14)	2.8 (0.72)	5.4

[1] = mean (SD); [2] = average feature prevalence relative to minimal expectance. Individual features can be part of several predictors to account for overlapping pathways. Thus, the total number of features and the number of unique features is given. Relative cluster prevalence indicates the recurrent selection of features for model generation. High values indicate high predictive relevance and stable contribution to model building (K = 5, R = 50).
