# Supplementary material for: Sexual epigenetics: gender-specific methylation of a gene in the sex determining region of Populus balsamifera
Source: Sci Rep. 2017 Mar 27;7:45388. doi: 10.1038/srep45388 (PMC5366940; doi:10.1038/srep45388)
Supplement: Supplementary Information [file srep45388-s1.pdf]

**SUPPLEMENTARY INFORMATION**

# Sexual epigenetics: gender-specific methylation of a gene in the sex determining region of *Populus balsamifera*

**Katharina Bräutigam[1], Raju Soolanayakanahally[2], Marc Champigny[3], Shawn Mansfield[4], Carl Douglas† [5], Malcolm M. Campbell[6], Quentin Cronk[5]**

[1]Department of Biology, University of Toronto, Mississauga ON, L5L 1C6, Canada
[2] Saskatoon Research and Development Centre, Agriculture and Agri-Food Canada, 107 Science Place, Saskatoon SK, S7N OX2, Canada.
[3] Department of Biological Sciences, University of Toronto Scarborough, Toronto, ON M1C 1A4, Canada
[4] Department of Wood Science, University of British Columbia, 4030-2424 Main Mall, Vancouver BC, V6T 1Z4, Canada
[5] Department of Botany, University of British Columbia, Vancouver BC, V6T 1Z4, Canada
[6] Department of Molecular and Cellular Biology, University of Guelph, Guelph ON N1G 2W1, Canada
*corresponding author: quentin.cronk@ubc.ca
† deceased

SUPPLEMENTARY TABLES AND FIGURE

**Table S1**. Poplar xylem samples used for statistical learning. Sample description including gender and assignment to either training or test data set is given (random genotypes = split 1; random samples = split 2). F: female, M: Male, Train: Training data set. Test: Test data set.

| Sample ID | Gender | Split 1 | Split 2 |
|---|---|---|---|
| BOY08_IHX | M | Train | Train |
| BOY08_PAX | M | Train | Train |
| BOY12_IHX | F | Train | Train |
| BOY12_PAX | F | Train | Train |
| FRE05_IHX | M | Test | Train |
| FRE05_PAX | M | Test | Train |
| FRE12_IHX | M | Train | Test |
| FRE12_PAX | M | Train | Train |
| LAR05_IHX | M | Train | Train |
| LAR05_PAX | M | Train | Test |
| LOV01_IHX | M | Train | Train |
| LOV01_PAX | M | Train | Test |
| LOV04_IHX | M | Train | Test |
| LOV04_PAX | M | Train | Train |
| LOV07_IHX | M | Test | Test |
| LOV07_PAX | M | Test | Test |
| POR04_IHX | F | Train | Test |
| POR04_PAX | F | Train | Train |
| POR05_IHX | M | Train | Train |
| POR06_IHX | M | Train | Train |
| POR06_PAX | M | Train | Train |
| POR06Rep_IHX | M | Train | Test |

| | | | |
|---|---|---|---|
| POR06Rep_PAX | M | Train | Train |
| POR12_IHX | M | Train | Train |
| POR14_IHX | F | Test | Train |
| POR14_PAX | F | Test | Test |
| ROS01_PAX | M | Train | Train |
| ROS01_IHX | M | Train | Train |
| ROS15_IHX | F | Train | Train |
| ROS15_PAX | F | Train | Train |
| SOU01_IHX | F | Train | Train |
| SOU01_PAX | F | Train | Train |
| SOU03_IHX | F | Test | Train |
| SOU03_PAX | F | Test | Train |
| SOU09_IHX | F | Train | Train |
| SOU09_PAX | F | Train | Test |
| WHR11_IHX | F | Train | Train |
| WHR11_PAX | F | Train | Train |
| WHR15_IHX | M | Test | Train |
| WHR15_PAX | M | Test | Train |
| WOL08_IHX | F | Test | Test |
| WOL08_PAX | F | Test | Test |

**Table S2.** Genome-wide methylation rate for different methylation contexts, percentages based on counts (in brackets based on averages).

| | **Total number of Cs studied (in brackets, the number of unique Cs studied)** | **Number methylated** | **% methylated, e.g. studied mCG/all studied CG. Based on counts (in brackets based on averages)** |
|---|---|---|---|
| CG contexts studied | 4607026008 (157538976) | 2501914313 | 54.3 (53.5) |
| CHG contexts studied | 5931965070 (218636981) | 2647348622 | 44.6 (40.5) |
| CHH contexts studied | 38592700632 (1382672499) | 3041784061 | 7.9 (8.0) |
| Total | 49,131,691,710 (1758848456) | 8191046996 | 16.7 (16.1) |

**Table S3.** Percentage contribution of different methylation contexts (CG, CHG, CHH) to total genome-wide methylated positions. Percentages are based on counts (based on averages in brackets).

| | **Number of Cs methylated** | **Different contexts (CG, CHG, CHH) as % of total methylated positions, based on counts (in brackets, based on averages)** |
|---|---|---|
| Methylated positions (CG) | 2501914313 | 30.5 (29.7) |
| Methylated positions (CHG) | 2647348622 | 32.3 (31.2) |
| Methylated positions (CHH) | 3041784061 | 37.1 (39.1) |
| Total methylated positions | 8191046996 | 100 |

**Table S4**. Parameter evaluation of the Pelora classifier (training set, split1). q: number of clusters, λ:penalty parameter. The average number of features that collectively comprise the predictors in PLG to model and predict gender is given along with the number of features per predictor and the misclassification rate in the training set (5 fold cross validation (K=5); 50 iterations). A misclassification rate of 0.033 corresponds to one misclassified sample.

| pelora model | # total input features CG context | # features in predictors[1] | # average features per cluster[1] | misclassification rate |
|---|---|---|---|---|
| q=2, λ=1 | 243,197 | 10.4 ± 3.4 | 5.2 ± 1.8 | 0.140 |
| q=2, λ=0 | 243,197 | 13.8 ± 3.3 | 6.9 ± 2.6 | 0.069 |
| q=2, λ=1/32 | 243,197 | 11.3 ± 3.1 | 5.7 ± 1.9 | 0.037 |
| q=4, λ=1/32 | 243,197 | 23.2 ± 5.3 | 5.1 ± 1.9 | 0.021 |
| q=6, λ=1/32 | 243,197 | 35.1 ± 7.3 | 5.8 ± 1.9 | 0.019 |
| q=8, λ=1/32 | 243,197 | 47.1 ± 9.2 | 5.9 ± 1.9 | 0.018 |
| **q=10, λ=1/32** | **243,197** | **59.54 ± 11.3** | **6.0 ± 1.9** | **0.016** |

**Figure S1**. Methylation in female and male samples for loci in the sex-determining region (SDR) in *Populus* as defined by Geraldes et al. (2015). In addition to the gene body, 2kb of upstream and downstream regions were also included. Methylation values are shown for tiles (500bp, coverage in 80% of samples). Data are row normalized and missing values are color coded in grey. The arrow marks the proximal promoter and beginning of PbRR9 (the only region showing clear sex-

specific methylation). Methylation patterns for contexts CG, CHG and CHH are shown separately.

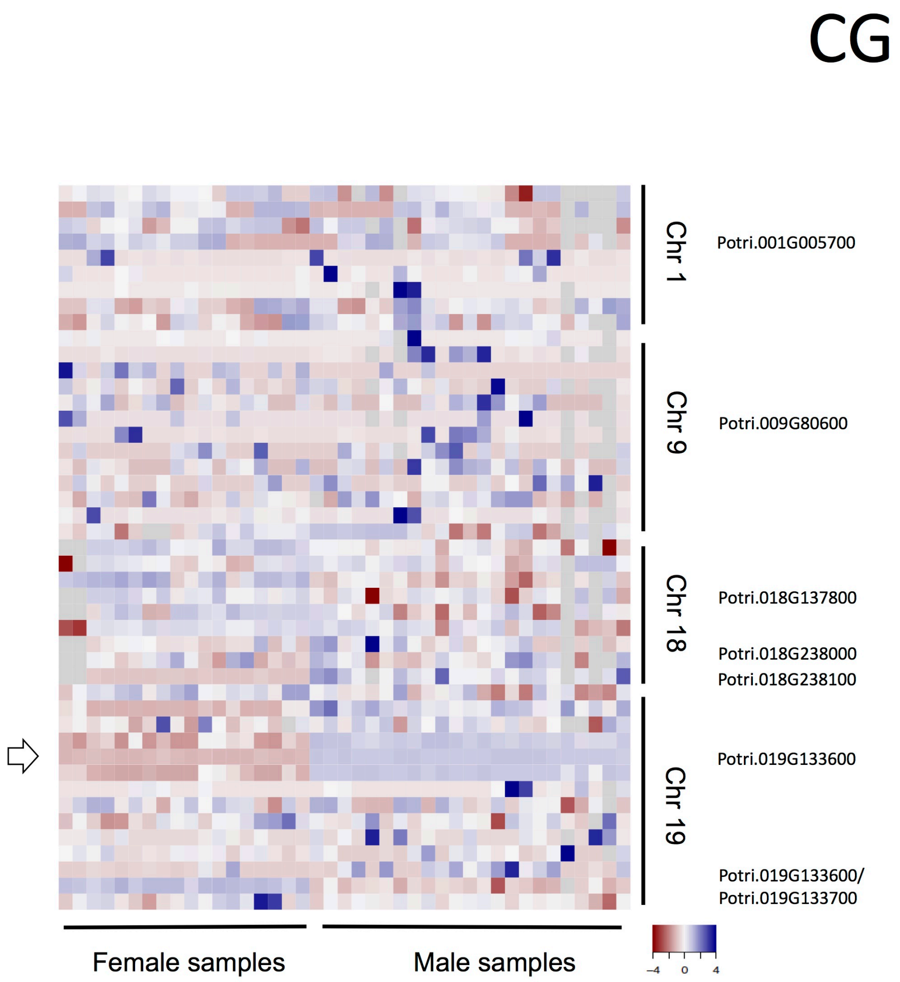

# CHG

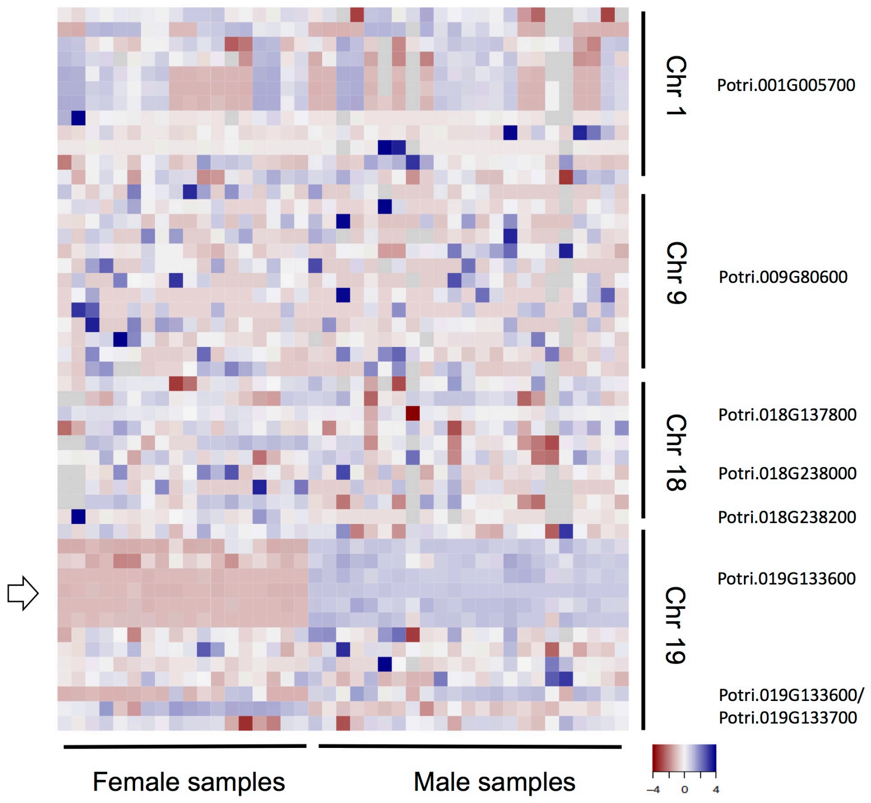

Chr 1
Potri.001G005700
Chr 9
Potri.009G80600
Chr 18
Potri.018G137800
Potri.018G238000
Potri.018G238200
Chr 19
Potri.019G133600
Potri.019G133600/
Potri.019G133700
Female samples
Male samples
−4
0
4

# CHH

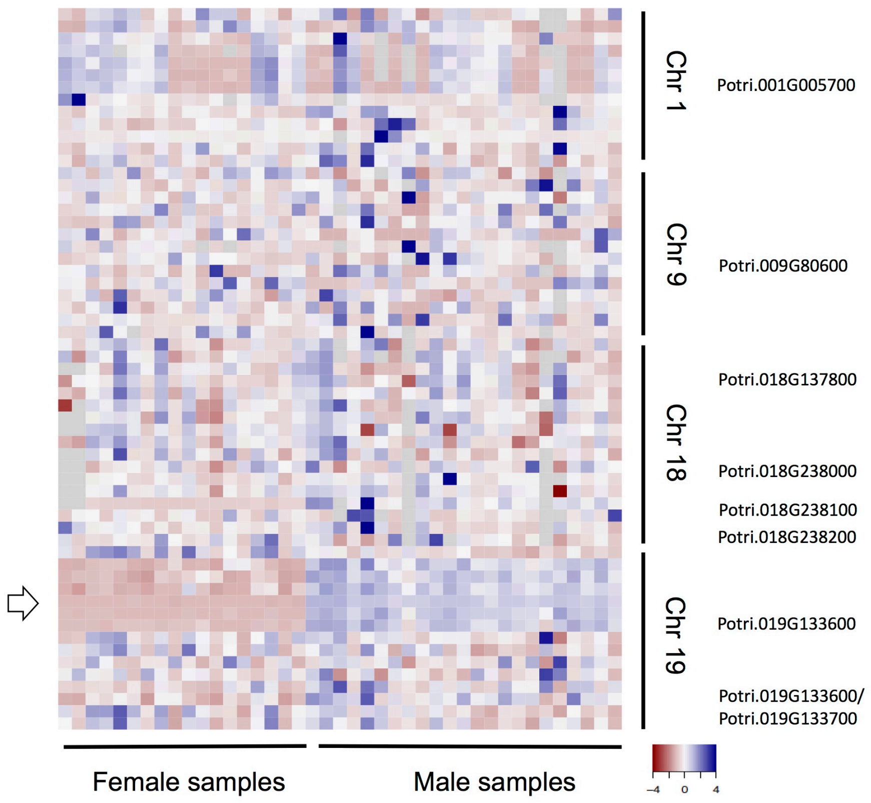

Chr 1
Potri.001G005700
Chr 9
Potri.009G80600
Chr 18
Potri.018G137800
Potri.018G238000
Potri.018G238100
Potri.018G238200
Chr 19
Potri.019G133600
Potri.019G133600/
Potri.019G133700
Female samples
Male samples
-4
0
4
